# Laser-induced speckle scatter patterns in *Bacillus* colonies

**DOI:** 10.3389/fmicb.2014.00537

**Published:** 2014-10-14

**Authors:** Huisung Kim, Atul K. Singh, Arun K. Bhunia, Euiwon Bae

**Affiliations:** ^1^Applied Optics Laboratory, School of Mechanical Engineering, Purdue UniversityWest Lafayette, IN, USA; ^2^Molecular Food Microbiology Laboratory, Department of Food Science, Purdue UniversityWest Lafayette, IN, USA

**Keywords:** bacterial colony, diffraction, speckle, phase modulation, *Bacillus*

## Abstract

Label-free bacterial colony phenotyping technology called BARDOT (Bacterial Rapid Detection using Optical scattering Technology) provided successful classification of several different bacteria at the genus, species, and serovar level. Recent experiments with colonies of *Bacillus* species provided strikingly different characteristics of elastic light scatter (ELS) patterns, which were comprised of random speckles compared to other bacteria, which are dominated by concentric rings and spokes. Since this laser-based optical sensor interrogates the whole volume of the colony, 3-D information of micro- and macro-structures are all encoded in the far-field scatter patterns. Here, we present a theoretical model explaining the underlying mechanism of the speckle formation by the colonies from *Bacillus* species. Except for *Bacillus polymyxa*, all *Bacillus* spp. produced random bright spots on the imaging plane, which presumably dependent on the cellular and molecular organization and content within the colony. Our scatter model-based analysis revealed that colony spread resulting in variable surface roughness can modify the wavefront of the scatter field. As the center diameter of the *Bacillus* spp. colony grew from 500 to 900 μm, average speckles area decreased two-fold and the number of small speckles increased seven-fold. In conclusion, as *Bacillus* colony grows, the average speckle size in the scatter pattern decreases and the number of smaller speckle increases due to the swarming growth characteristics of bacteria within the colony.

## Introduction

Bacterial colonies consist of millions of individual cells interacting with each other in complex ways (Shapiro, 1992; Wimpenny, 1992; Shimada et al., 1995; Ben-Jacob et al., 1998). It is important to understand the mechanism of colony formation and the resulting morphology as it is a key to understand organizations and interactions among bacterial cells within a colony. The colony morphology is highly influenced by the shape of individual cells, cell wall components (peptidoglycan, teichoic acid, lipopolysaccharide), the extracellular components and appendages (flagella, fimbriae, curli), and cellular response to the environmental cues including nutrient availability, oxygen and other gases, salt, acidity, alkalinity, temperature, etc. (Nagai et al., 1971; Granek and Magwene, 2010). Therefore, the colony morphology of different bacteria could be used as a foundation for differential characterization using an interrogating tool.

Historically, bacterial colony pattern formation has drawn significant interest among diverse research fields. Microbiologists were more interested in determining how bacterial response to the environmental factors such as nutrient and agar hardness affect colony formation, while the physicist were aiming to recreate the colony formation scenario by using theoretical models. Shapiro and Wimpenny first observed bacterial colony formation in time and space (Shapiro, 1992; Wimpenny, 1992) and since then, many other researchers employed both experimental (Shimada et al., 1995; Ben-Jacob et al., 1998; Bees et al., 2000; Stecchini et al., 2001; Kaito and Sekimizu, 2007; Pipe and Grimson, 2008) and theoretical models (Kawasaki et al., 1997; Cohen et al., 1999; Kozlovsky et al., 1999; Lega and Passot, 2003) to explain such phenomenon. Among the various organisms tested, a majority of the study used *Bacillus* spp. as a model due to their swarming growth characteristics. Swarming colonies can generate diverse spatio-temporal patterns due to their reproduction and spreading mechanisms (Harshey, 2003). Therefore, we also studied *Bacillus* as a model organism to correlate the swarming colony morphology to the optical light scattering and speckle effect.

Since the first reports (Bae et al., 2007; Banada et al., 2007), optical light scattering of colony have expanded to differentiate diverse bacterial genera. The remarkable resolving power of the BARDOT originates from the accumulation and amplification of both microscopic structural and biochemical differences that exist among different bacterial colonies through an interrogating laser beam. When the laser beam passes through the bacterial colony, both 3D morphological and optical characteristics are integrated into 2D outgoing wave and encoding it on the coherent optical wavefront. The wavefront then propagates through near- and far-fields governed by the diffraction integral to form distinctive forward scattering pattern which serves as an optical “fingerprint.”

Elastic light scatter (ELS) has been used by our group for bacterial colony differentiation and identification (Bae et al., 2007, 2011; Banada et al., 2009; Huff et al., 2012; Singh et al., 2014). Recently we have used this technology for differentiating *Bacillus cereus* (an important food poisoning species) and compared its colony scattering pattern with another ubiquitous species, *Escherichia coli* (Singh et al., submitted). *Bacillus* is highly motile and considered a swarming bacterium, which shows greater mobility on solid agar. Among the *Bacillus* spp., surprisingly, only *B. polymyxa* expressed typical ELS patterns of concentric rings and spokes consistent with other bacterial genera: *Listeria*, *Salmonella*, *Vibrio, Escherichia*, and *Staphylococcus* (Bae et al., 2007; Banada et al., 2009; Huff et al., 2012; Singh et al., 2014). While all other *Bacillus* species tested showed random speckles overlaid with some circular ring patterns (Singh et al., submitted). The scattering patterns of *B. subtilis* and *B. polymyxa* have shown significant differentiating characteristics. In this paper, we investigated the different optical properties of these two *Bacillus* species; (i) to understand how *B. subtilis* and *B. polymyxa* construct their colony using recently developed Integrated Colony Morphology Analyzer (ICMA) (Kim et al., 2013); (ii) to compare the theoretical prediction of ELS patterns with experimental data; and (iii) to calculate the speckle statistics to quantitatively correlate the optical phase modulation into the structure of the bacterial colony.

## Materials and methods

### Sample preparation

*Bacillus subtilis* ATCC 6633 (*B. subtilis*)*, Bacillus polymyxa* B719W (*B. polymyxa*), *Bacillus cereus* ATCC 14579 (*B. cereus*), and *Bacillus thuringiensis* DUP6044 (*B. thuringiensis*) cultures were used in this study. Cultures were inoculated in brain heart infusion (BHI) broth (Difco, MD, USA) and incubated at 37°C for overnight (16 h) with 130 rpm shaker, 10-fold serially diluted in 0.2 mM phosphate buffered saline, pH 7.0 (PBS) and diluents were plated on BHI and phenol red mannitol (PRM) agar plates (Becton Dickinson, NJ, USA) to obtain 50–100 colonies. Optical scatter patterns of the colonies were captured using the BARDOT instrument at 6–8 h that corresponds to colony diameter of about 550, 750, and 900 μm, respectively.

### Forward scatterometer

**Figure 1:**
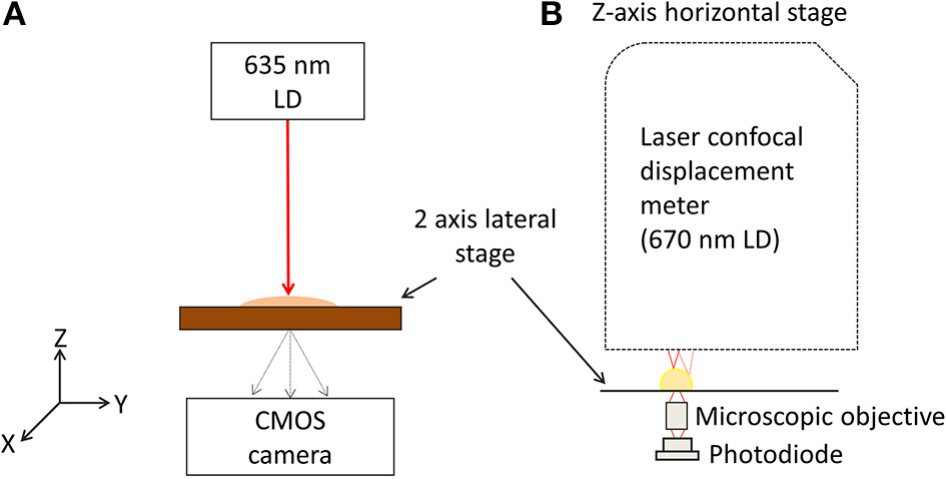
**Schematic diagram of the experimental setup. (A)** Schematic diagram of BARDOT instrument, laser diode (LD) with 635 nm wavelength is used as a light source and directly impinging the single bacterial colony grown on an agar plate. Spatially scattered light is captured by a 2-D detector. **(B)** Schematic diagram of ICMA, *in-situ* morphology and optical density map of bacterial colony measurement unit.

Scatterometer (Figure 1A), consists of laser diode (LD) with 635 nm wavelength that was installed as a light source, and the light source directly illuminates a single bacterial colony grown on semi-solid agar plate. The diffracted light is captured using detector (CMOS camera, PL-B741, ON, Canada) with 1280(H) × 1024(V) pixels, 6.7 × 6.7 μm pixel pitch which is located after the semi-solid agar plate with distance z. Motorized 2 axis lateral stage was integrated to the system, and the semi-solid agar plate was put on the 2 axis lateral stage for a full automatic measurement of optical scatter pattern. The *Bacillus* colonies were then picked from the plate, grown briefly (4 h) in BHI broth at 37°C and analyzed by multiplex PCR (mPCR) assay.

### DNA extraction and mPCR

Total DNA was extracted by boiling cultures as described earlier (Ngamwongsatit et al., 2008). mPCR was performed using *gyrB* gene-specific primers (BcF: 5′ GTTTCTGGTGGT TTACATGG3′; BcR: 5′TTTTGAGCGATTTAAATGC 3′) and *cry* gene-specific primers (K5F: 5′AGG ACCAGGATTTACAGGAGG 3′; K3R: 5′ GCTGTGACACGAAGGATATAGCCAC 3′) (Kuo and Chak, 1996; Manzano et al., 2003). The mPCR reaction mixture contained 200 μM of each dNTP, 2.5 mM of MgCl_2_, 0.8X GoTaq Flexi buffer, 1U of GoTaq Flexi DNA polymerase (Promega), 0.2–0.3 μM of primers, 60–90 ng of template DNA and the reaction condition was optimized (Henegariu et al., 1997; Lorenz, 2012). Ultra-pure sterile water was used as negative control. PCR reaction was only considered valid when control reaction was either positive or negative as appropriate and 16S rRNA gene was positively amplified with all DNA templates. All amplicons were analyzed on 1.2% agarose gel.

### Integrated colony morphology analyzer (ICMA)

As shown in Figure 1B, the laser confocal displacement meter (CDM) (Keyence, LT9010M) is located at the top and attached to a linear horizontal translation stage (Edmund Optics, NJ, USA) in order to position the probing laser beam (λ = 670 nm) along the vertical z axis and to focus it on the surface of colony. A motorized 2D lateral stage translated the Petri dish in the x-y plane to align the diagnostic laser beam with each selected bacterial colony to obtain their 3-D profiles. Two linear motors (850G-HS) connected to the ESP 300 multi-axis closed-loop controller (Newport, NY, USA) with the specification of a 42 mm maximum stroke and a 0.1 mm minimum step size control the movement of the x-y stage. This instrument implements a high accuracy surface scanning method by using a laser light source with a Gaussian beam spot of approximately 2 μm. The position of the doublet lenses is controlled by a tuning fork moving up and down at high frequency. When the beam was focused on the sample (colony) surface, the reflected light traveled back along the original optical path and was directed toward the pinhole to produce the highest level of intensity on the light receiving element. If the laser is unfocused, some of the reflected light would be occluded at the pinhole. Therefore, the relative height of the measured surface was registered by the sensor positioned at the bottom of the tuning fork, and the values are calculated and converted to an absolute height value (Keyence-Corporation, 2006).

A micro-objective lens (Bausch & Lomb, 20x, N.A. 0.4) is placed just below the Petri dish to focus the transmitted light onto the active area of the photodiode (Hamamatsu, S1087) (Figure 1B). This photodiode offered a low background noise (10 pA) with a wide dynamic range and provided a spectral response from 320 to 730 nm with the peak sensitivity at 560 nm. The active area for this photodiode is 1.3 × 1.3 mm^2^, providing a photo sensitivity of 0.19 A/W for the incoming laser from the CDM sensor head on which a custom built active components driven I-V converter and preamp were installed at a preamp section. A custom built micro controller unit (MCU) (AVR128, Atmel) was used as a data acquisition unit. Through use of the MCU's internal 10 bit A/D conversion, all signals from the photodiode circuit and CDM were captured and transferred simultaneously to the PC by means of serial communication. All sequences were controlled by a custom built graphic user interface (GUI) at the PC, which was developed using Microsoft Visual Studio 2008 and both were analyzed and visualized in MATLAB.

### Modeling speckle effect from elastic light scattering

The bacterial colony and a semi-solid media are positioned at the aperture plane, and the forward scattering pattern is captured at the image plane, defined as (x_a_, y_a_) and (x_i_, y_i_), respectively. A bacterial colony is modeled as a bell curve shape with tailing edge (Gaussian-like profile) where colony center height and radius is defined as *H*_0_ and *r*_*c*_, respectively. Based on Rayleigh and Sommerfeld formation of diffraction, and Fresnel diffraction approximation (Bae et al., 2007, 2010), intensity of electric field at aperture and image plane is derived as Equations (1) and (2), respectively, where distance between aperture plane to a point at image plane, *r*_*ai*_ is assumed as Equation (3).

(1) $$\begin{array}{l} E_{a}(x_{a},y_{a},z)=E_{0}\exp\left[-\frac{\left(x_{a}^{2}+y_{a}^{2}\right)}{\omega^{2}(z)}\right]\exp(ikz)\\ \;\exp\left[ik\frac{\left(x_{a}^{2}+y_{a}^{2}\right)}{2R(z)}\right] \end{array}$$

(2) $$\begin{array}{l} E_{i}(x_{i},y_{i})=\frac{1}{i\lambda }\iint t(x_{a},y_{a})E_{a}(x_{a},y_{a})\exp\left[ik\Phi (x_{a},y_{a})\right]\\ \;\frac{\exp\left[ikr_{ai}\right]}{r_{ai}}\cos\theta \text{d}x_{a}\text{d}y_{a} \end{array}$$

(3) $$\begin{array}{l} r_{ai}={\left[z_{i}^{2}+{\left(x_{a}-x_{i}\right)}^{2}+{\left(y_{a}-y_{i}\right)}^{2}\right]}^{\frac{1}{2}}\\ \;≅z_{i}\left[1+\frac{1}{2}{\left(\frac{x_{a}-x_{i}}{z_{i}}\right)}^{2}+\frac{1}{2}{\left(\frac{y_{a}-y_{i}}{z_{i}}\right)}^{2}\right] \end{array}$$

**Figure 2:**
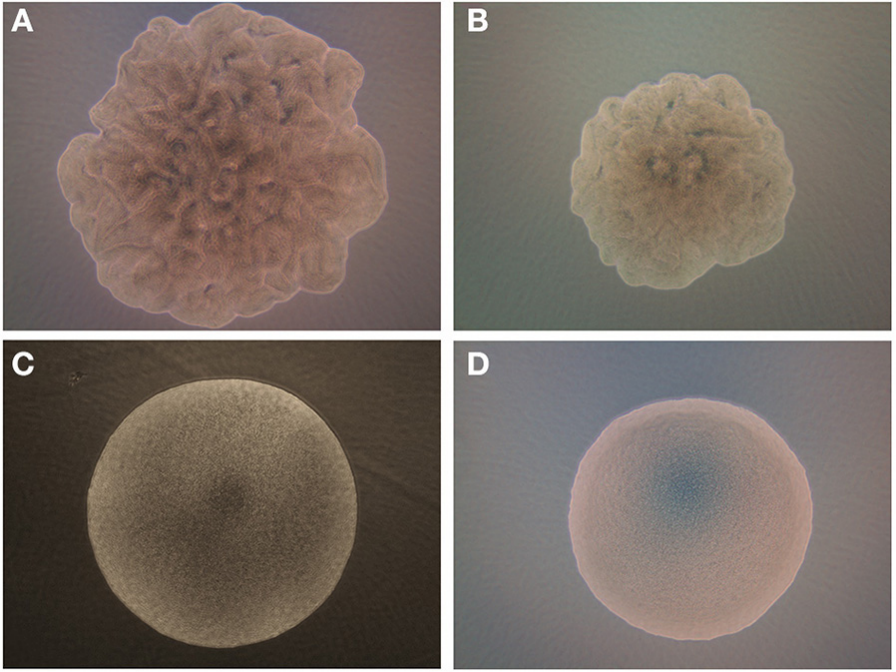
**Phase contrast images of the *B. subtilis* on (A) BHI and (B) PRM where colony shows swarming growth characteristics**. For comparison, *B. polymyxa* on **(C)** BHI and **(D)** PRM are shown where colony forms a circular shape.

**Figure 3:**
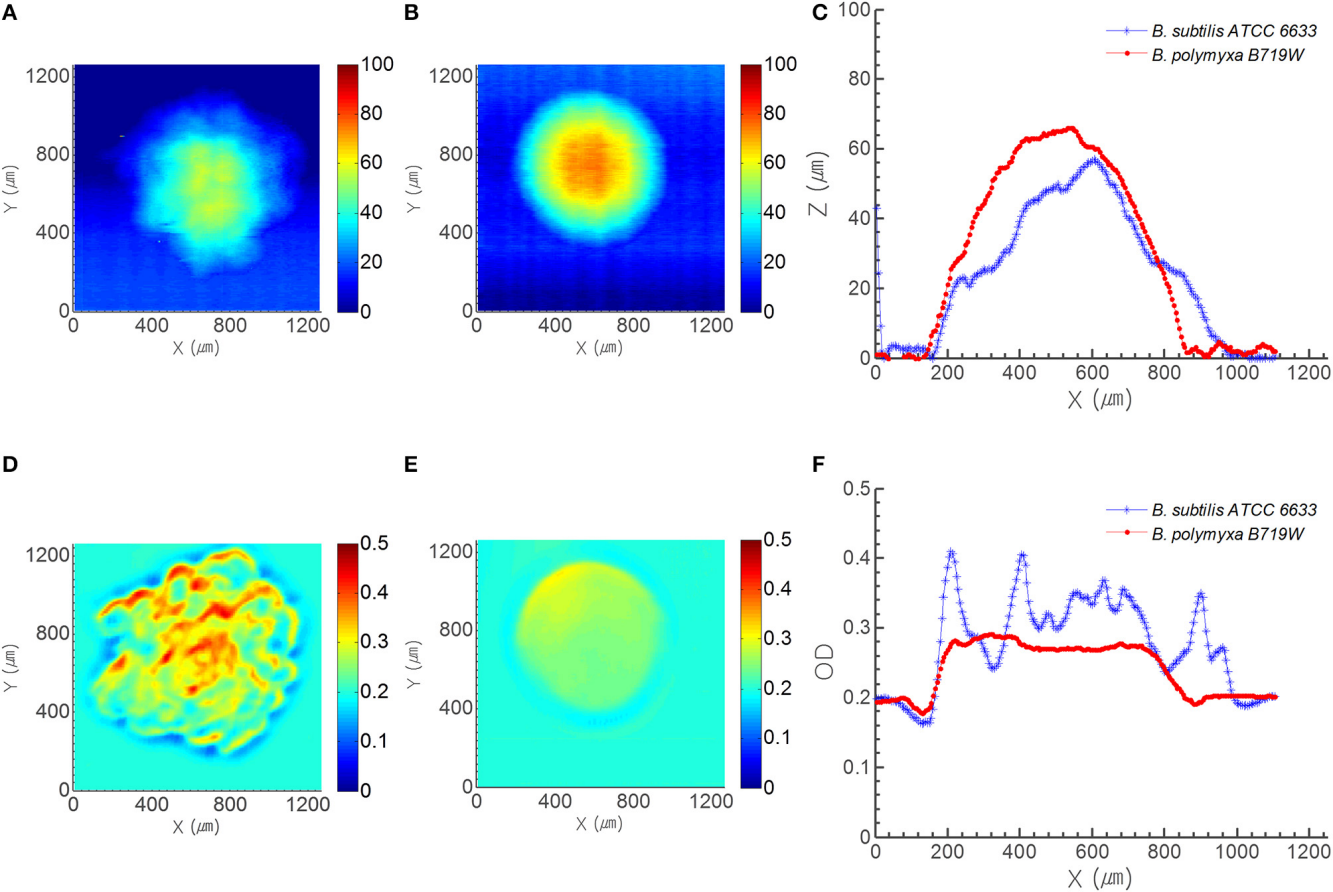
**Profile and OD map acquired from ICMA**. Top row displays the profile map of **(A)** *B. subtilis* ATCC 6633 colony and **(B)** *B. polymyxa* B719W colony, and **(C)** profile cross section of both species. Bottom row shows the OD map of **(D)** *B. subtilis* ATCC 6633 colony and **(E)** *B. polymyxa* B719W colony, and **(F)** OD cross section of both species on PRM.

Since previous researchers focused on structural morphology of bacterial colony such as elevation and colony diameter, the colony was modeled as smooth curve with Gaussian profile function without considering surface roughness. In reality, a cross section of bacterial colony showed the accumulation of densely packed multiple layers of bacterial cells (Banada et al., 2009; Bae et al., 2010; Suchwalko et al., 2013; Marcoux et al., 2014). Furthermore, *Bacillus* colonies also showed some degree of surface roughness (Figures 2, 3). To simplify the modeling, surface roughness is generated by random signal with colony area, and superposed to the colony morphology. Considering the surface roughness of the colony, intensity of electric field at image plane is simplified as Equation (4).

(4) $$\begin{array}{l} E_{i}(x_{i},y_{i})=C\iint \text{T}(x_{a},y_{a})\exp\left[i\Phi_{r}\right]\exp\left[i\Phi_{c}\right]\exp\left[i\left(\Phi_{g}+\Phi_{s}\right)\right]\\ \;\exp\left[-2\pi i(f_{x}x_{a}+f_{y}y_{a})\right]\text{d}x_{a}\text{d}y_{a} \end{array}$$

where T is amplitude modulator; *f_x_* and *f_y_* is defined as *xi*/(λ*z*_2_) and *yi*/(λ*z*_2_), known as a spatial Fourier frequency; Φ_*r*_, Φ_*q*_, Φ_*g*_, and Φ_*s*_ is radial, quadratic, Gaussian, and surface roughness phase component respectively, and defined as:

(5) $$\Phi_{r}(x_{a},y_{a})=\frac{k\left(x_{a}^{2}+y_{a}^{2}\right)}{2R}$$

(6) $$\Phi_{q}(x_{a},y_{a})=\frac{k\left(x_{a}^{2}+y_{a}^{2}\right)}{2z_{i}}$$

(7) $$\Phi_{g}(x_{a},y_{a})=k(n_{bac}-1)H_{0}\exp\left[-\frac{\left(x_{a}^{2}+y_{a}^{2}\right)}{r_{c}^{2}}\right]$$

(8) $$\Phi_{s}(x_{a},y_{a})=k\left(\text{random}(x_{a},y_{a})\right)\text{ where }\left(x_{a}^{2}+y_{a}^{2}<r_{c}^{2}\right)$$

The summation of four phase components,Φ_*overall*_ is working as phase modulator for the propagating light. The amplitude modulator T is derived as:

(9) $$\begin{array}{l} T(x_{a},y_{a})=\exp\left[-\frac{\left(x_{a}^{2}+y_{a}^{2}\right)}{\omega^{2}(z)}\right]\frac{E_{out}}{E_{0}}\\ \;=\exp\left[-\frac{\left(x_{a}^{2}+y_{a}^{2}\right)}{\omega^{2}(z)}\right]\left(1-r_{air-bac}\right){\left(1-r_{k}\right)}^{2l}\\ \;\left(1-r_{bac-agar}\right) \end{array}$$

(10) $$\Phi_{overall}=\Phi_{r}+\Phi_{q}+\left(\Phi_{g}+\Phi_{s}\right)$$

Coefficient of Equation (4), C is derived as:

(11) $$C=\frac{\begin{array}{c} E_{0}\exp(ikn_{agar}\Delta_{agar})\exp(ikH_{0})\exp\left[ik(z+z_{i})\right]\\ \;\exp\left[ik(x_{i}^{2}+y_{i}^{2})/2z_{i}\right] \end{array}}{i\lambda z_{i}}$$

where Δ_agar_ and *n*_agar_ is defined as thickness of agar and refractive index of agar respectively.

### Speckle analysis

To provide quantitative speckle analysis, a MATLAB code was constructed utilizing an image processing toolbox V2.6 (Vliet, 2014). A radial Gaussian blur was applied to the raw image to apply a low pass filter that eliminates the objects while keeps the slow change in the background, and the blurred image was subtracted from the raw image to remove the background noise. The subtracted image was binarized with locally adaptive threshold using approximation of gradient of background intensity to eliminate the external lighting and local CMOS sensor sensitivity characteristics effect on the image. Then, each speckle was segmented, grouped, and labeled. Number of pixels (size of the speckle) and feret diameter (Merkus, 2009) of each segmented group was computed, and analyzed.

## Results

Here, we report how colony morphology correlates with its speckle patterns using both theoretical and experimental approaches. Thus, each colony was first interrogated by ICMA and phase contrast microscopy to record height, transmittance and morphology.

### Colony development profiles

The phase contrast microscopic (PCM) images of colonies of *B. subtilis* and *B. polymyxa* on both BHI and PRM were contrasting (Figure 2). *B. subtilis* colony has an irregular boundary with bumpy and uneven surface structure on both media (Figures 2A,B). Due to the limitation of phase contrast microscopic setup, the 3-D elevation cannot be interpreted with current image but *B. subtilis* shows a representative swarming colony characteristics, where spatially localized variations of bacterial cell densities are observed. Meanwhile, *B. polymyxa* had symmetrical circular boundary with relatively smooth surface curvature (Figures 2C,D). As the PCM result showed, the colony forming characteristics of *B. polymyxa* was drastically different from the majority of other *Bacillus* species including *B. subtilis, B. cereus*, and *B. thuringiensis*.

### Comparison of transmittance and elevation profile

To compensate the phase contrast microscope image, ICAM was utilized to acquire spatially resolved optical characteristics from a single colony. Figures 3A,B show the morphology map of *B. subtilis* and *B. polymyxa* colony on PRM, respectively from morphology channel of ICMA; whereas panel (d) and (e) show their corresponding spatially resolved transmittance in optical density (OD) units with 670 nm light source. Colonies of both species on PRM agar were captured after 7 h of growth, when the center diameter of *B. polymyxa* colony reached to approximately 800 μm. Similar to the PCM result, morphology map by ICMA revealed that boundary of *B. subtilis* colony was irregular, while, *B. polymyxa* colony had near circular symmetrical boundary (Figures 3A,B). Comparison of morphology cross section for the both species at their center area showed that *B. subtilis* colony had random bumpy profile with rough surface, while *B. polymyxa* colony had bell curve-like profile with relatively smooth surface (Figure 3C). *B. subtilis* colony had approximate 890 μm cross sectional diameter and 57 μm of center peak height, while, *B. polymyxa* colony had 814 μm diameter and 66 μm of colony center height. Considering the colony aspect ratio (colony center height to diameter ratio) of each species, *B. subtilis* (1:15.6) colony had more flat cross sectional profile than *B. polymyxa* (1:12.4) at the same diameter. Furthermore, the 2-D transmittance map (cross-sectional OD profile) of both species shows more distinctive characteristics, which can be attributed to colony morphology and opacity (Figures 3C,F). Since the OD of BHI agar plate alone at 670 nm was approximately 0.2, cross section of the OD result had 0.2 offset. Cross section of *B. subtilis* colony OD fluctuates from 0.24 to 0.41, while *B. polymyxa* colony had OD with maxima 0.29. Assuming that each species of bacterial cell had identical optical characteristic, this irregularity and randomness of OD can be ascribed to different spatial cell density that is translated into variation in light absorption. In addition, surface roughness of the *B. subtilis* colony can be modeled as variation in reflected and transmitted light intensity (Figure 3).

### ELS measurement

**Figure 4:**
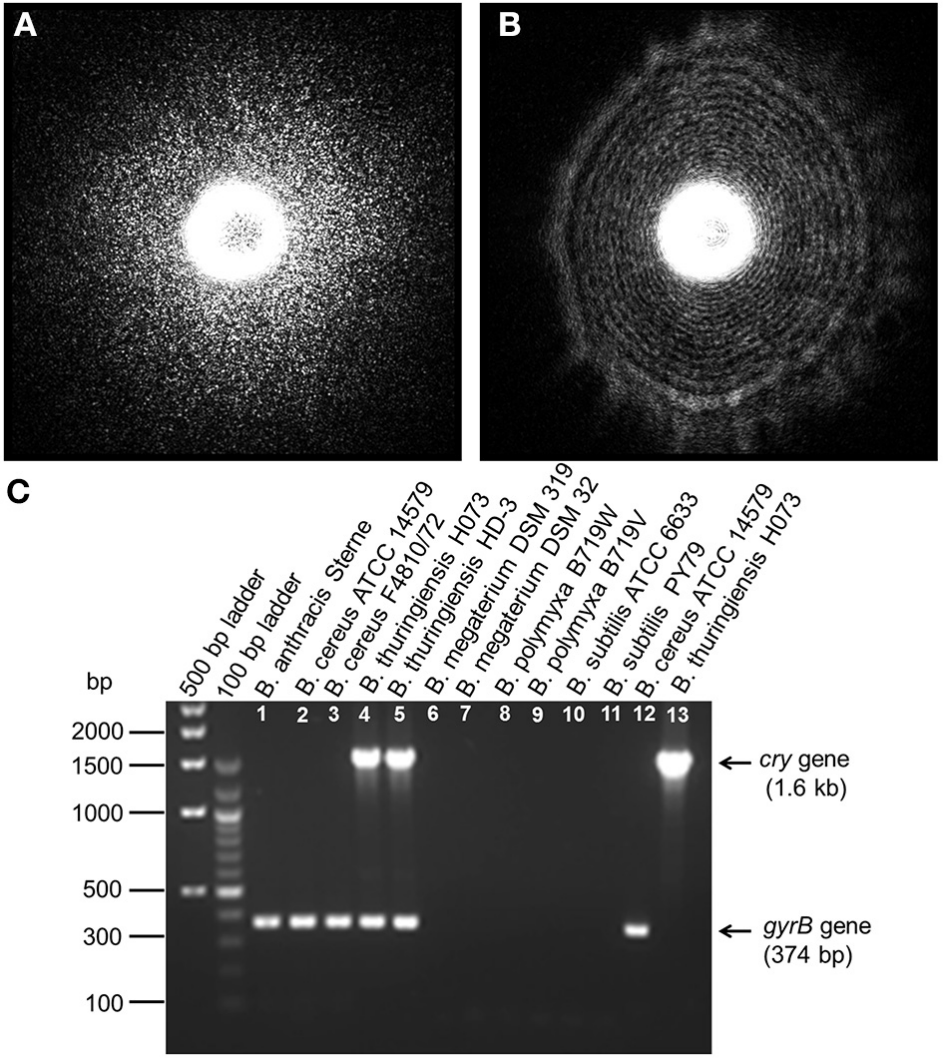
**Elastic light scatter (ELS) patterns on PRM agar for (A) *B. subtilis* ATCC 6633, and (B) *B. polymyxa* B719W; (C) Multiplex PCR (mPCR) for differentiation of *Bacillus cereus*-group strains (Lane 1–5) from other *Bacillus* species (Lane 6–11)**. mPCR resulted a 374 bp PCR product for all *Bacillus* cereus-group strains and a 1.6 kb size PCR product only for *B. thuringiensis*. Lane 12 and 13 represents single gene specific amplification for *gyraseB* gene in *Bacillus cereus* and *cry* gene in *Bacillus thuringiensis*. mPCR was performed with *gyrase B* gene-specific and cry protein gene-specific primers.

**Figure 5:**
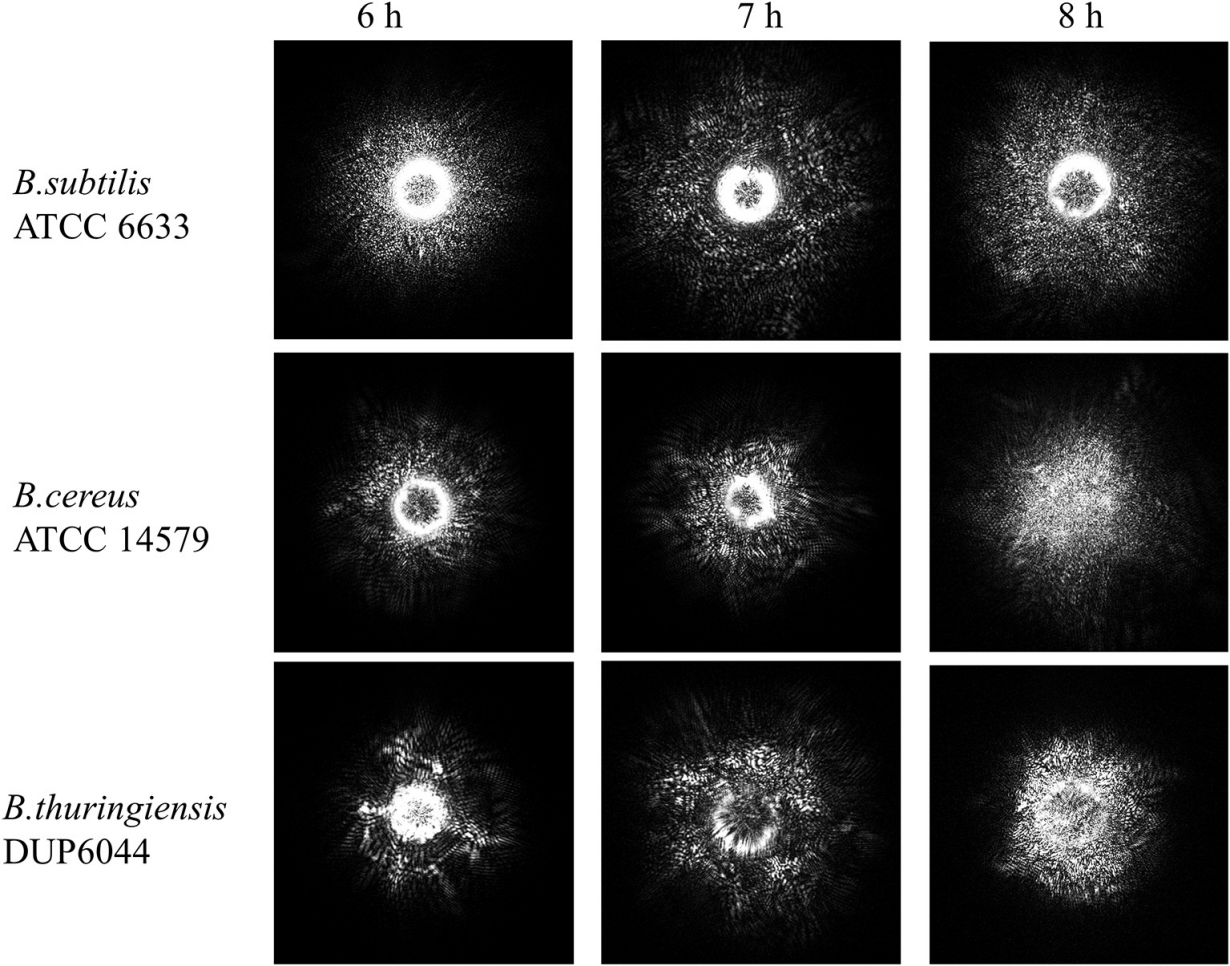
**Time-resolve ELS patterns for three different *Bacillus* species: *B. subtilis*, *B. cereus*, and *B. thuringiensis* on PRM agar**. As the incubation time increases from 6 to 8 h, ELS pattern evolves to a fully developed speckle when an average speckle diameter decreases.

In previous studies, optical forward scattering pattern or ELS of *Salmonella*, *E. coli*, and *Staphylococcus* showed characteristics symmetric or concentric circle with some radial spokes (Banada et al., 2009). However, forward scattering pattern of *Bacillus* species (except *B. polymyxa*) showed speckle pattern (Figures 4A, 5). Diffraction pattern of *B. subtilis* colony is consisted of only small sized random speckle, while, *B. polymyxa* shows concentric circular pattern (Figure 4). To verify the time resolved speckle development of *Bacillus* species on their forward scattering pattern, *B. subtilis, B. cereus*, and *B. thuringiensis* were selected and their diffraction patterns were measured after 6–8 h of growth using BARDOT (Figure 5). As the incubation time increased from 6 to 8 h, the diffraction patterns also evolved to fully developed speckles, i.e., structured patterns progressively became unstructured random speckles. All three *Bacillus* species displayed similar trend as the incubation time increased where average speckle numbers decreased.

### Comparative analysis of experiment with simulation

**Figure 6:**
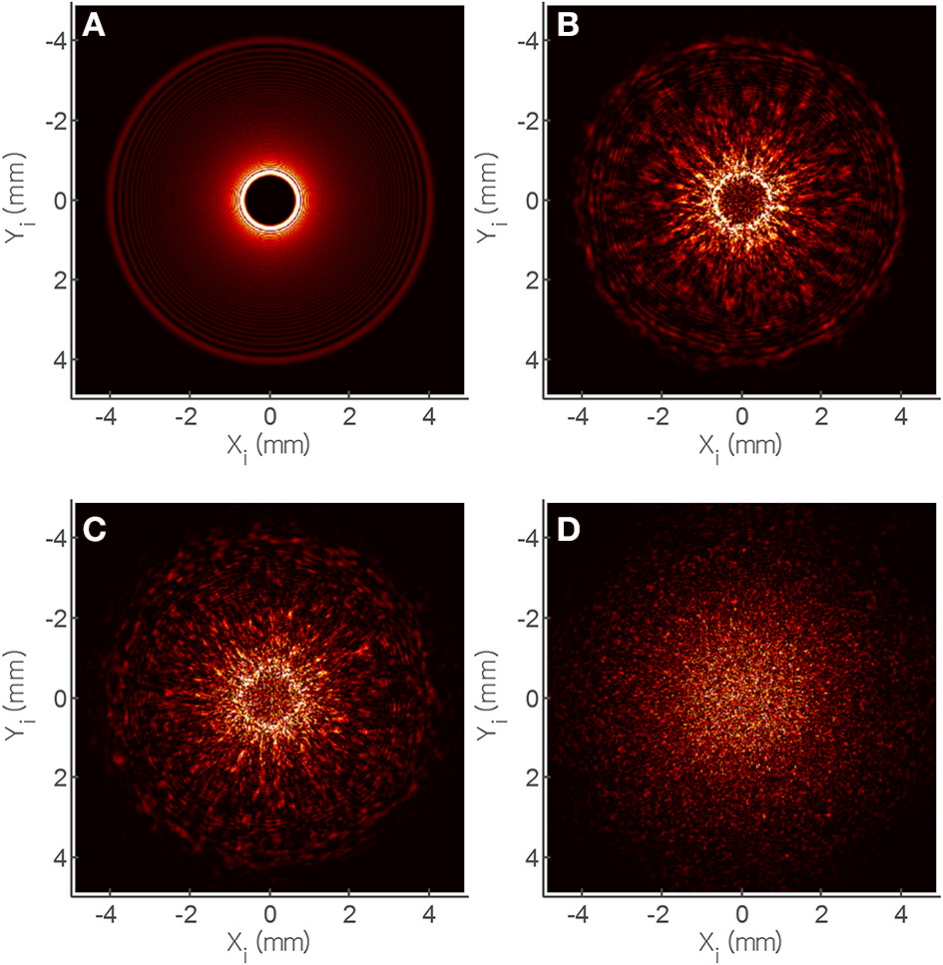
**Theoretical analysis of speckle development based on the random phase shift (φ_*s*_) of scalar diffraction theory**. Phase shift was modified as **(A)** 1/200, **(B)** 1/150, **(C)** 1/100, and **(D)** 1/80 and their simulated scatter patterns show similar trend with the experiment.

To investigate the effect of surface roughness on forward scattering pattern, other components of models are kept constant for Equations (5)–(11). The effect of random surface phase component (Φ_*s*_) on forward scattering pattern was determined (Figure 6). Figure 6A shows the forward scattering pattern prediction when the surface phase component was equal to zero which means, the colony surface was devoid of any random structures. The predicted scatter image contained concentric ring patterns with small bright ring at the center and the thickness of rings increased as they moved outwardly. To mimic the random surface roughness effect, Equation (8) was adopted which is a random function multiplied by the wave number *k*, and is only influenced by bacterial colony region. The maximum amplitude of the random function for Equation (8) was chosen as 1/150, 1/100, and 1/80 of colony center height for fine, medium, and coarse surface roughness, respectively. The surface roughness component (Φ_*s*_) is added to Gaussian phase component (Φ_*g*_), and worked as a part of phase modulator, which can deform the incident wave front. Predicted forward scatter patterns are shown for fine (Figure 6B), medium (Figure 6C), and coarse surface roughness (Figure 6D). For a diffraction pattern prediction with fine surface roughness effect, relatively larger size speckles and less number of speckles were generated, and still had concentric circle pattern. As the surface roughness increased, the size of the speckle decreased and higher number of speckles were generated, and formed a random speckle pattern rather than concentric circle pattern as predicted.

**Figure 7:**
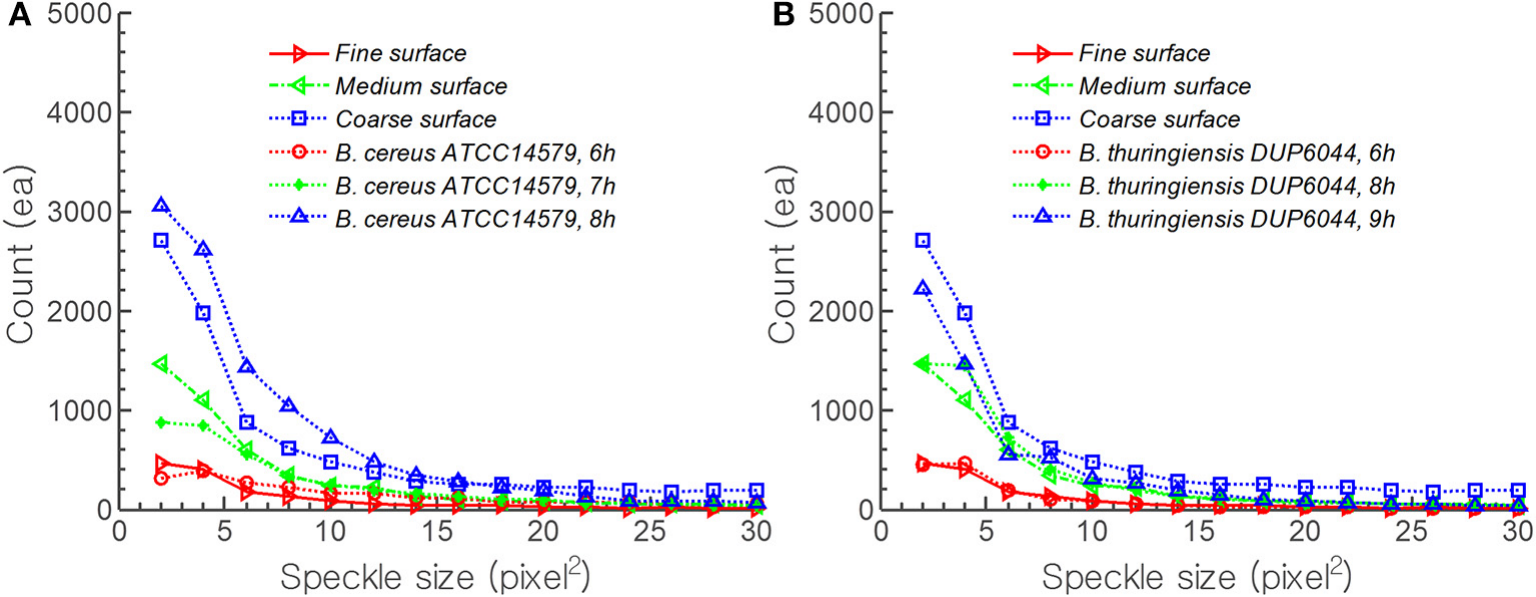
**Comparison of theoretical speckle development on diffraction pattern by surface roughness and that of experimental time resolved result for *B. cereus* and *B. thuringiensis***. For prediction, the maximum amplitude of random function of surface roughness was adopted as **(A)** 1/200, 1/150, and 1/100 **(B)** 1/180, 1/130, and 1/100 of colony center height for fine, medium, and coarse surface roughness, respectively.

Figure 7 shows the comparison of theoretical speckle development on diffraction pattern by surface roughness and that from experiment. The results were plotted for speckle area vs. the number of speckles. For the prediction, the maximum amplitude of the random function for Φ_*s*_ were set as 1/200, 1/150, and 1/100 of *B. cereus* colony center height for fine, medium, and coarse roughness, respectively. As the theoretical model moved from coarse (□) to fine (⊳) surface roughness, the model predicts decreasing average speckle size with increasing numbers. Similar trend was observed in experiment with *B. cereus* ATCC14579 incubated from 6 h (□) to 8 h (Δ) (Figure 7A). For *B. thuringiensis* DUP6044, Φ_*s*_ was changed to 1/180, 1/130, and 1/100 of colony center height and an excellent agreement between theory and experiment was evident (Figure 7B). For example, coarse surface roughness (□) and 9 h incubation (Δ) showed almost similar trend in average speckle size and the numbers.

## Discussion

Understanding bacterial growth on a solid surface is important for biophysicist, microbial ecologist, and food microbiologist for better insight of colony development and its ecological and virulence properties. In context to virulence attributes of colony, small colony variants (SCVs) of different bacteria are often considered more virulent facilitating persistence and recurrent infections (Proctor et al., 2006). Harshey (2003) wrote an excellent review on how bacteria form colony in four different ways (swarming, gliding, twitching, and sliding). Our motivation to this study was to correlate the unique growth characteristics of *Bacillus* species to the optical speckle theory and help understand colony growth dynamics. *Bacillus* has long been used as a model to study the environment dependent variations in growth patterns (Shimada et al., 1995; Ben-Jacob et al., 1998; Bees et al., 2000; Stecchini et al., 2001; Kaito and Sekimizu, 2007; Pipe and Grimson, 2008). However, recent application of optical scattering-based bacterial colony interrogation requires different perspective for fundamental understanding of scatter pattern generation and colony morphology.

Previous studies have shown that availability of nutrients and agar concentration affect the distribution/propagation of cells within a colony (Bae et al., 2011). However, the published studies on colony scatter pattern did not report theoretical interpretation of speckle pattern formation by a colony. To the best of our knowledge, this is the first report of theoretical interpretation of speckle pattern formation by *Bacillus* colony, which is largely attributed to the roughness of bacterial colony. The surface roughness is believed to be arisen from the swarming behavior of the *Bacillus* cells during colony formation. ICMA and PCM measurements revealed that *B. subtilis* generates more rough surface morphology than the *B. polymyxa* (Figures 2, 3).

For theoretical comparison, ICMA measurement was critical since the scalar diffraction theory required accurate description of the amplitude and phase modulation component of Equations (1)–(11). Both qualitative trend and quantitative measurement showed good agreement (Figure 7). Both theoretical model and the experimental evidence indicate that as bacteria grow, the speckle size decreases and the number increases. Furthermore, the quantitative comparison also revealed that as surface roughness changed from fine to coarse, the number of speckle increased 7 (*B. cereus*) to 10 (*B. thuringiensis*) -fold. Similar quantitative trend was observed for two other bacterial species: *B. cereus* and *B. thuringiensis*. In future, this information could be used to provide fundamental understanding of inverse scattering method for analyzing bacterial colony formation since size and the number of speckle correlate with incubation time and surface roughness.

The light scattering sensor discussed in this study was successfully used for detection of bacterial pathogens including *Listeria monocytogenes* (Banada et al., 2007, 2009), *Vibrio* spp. (Huff et al., 2012), *Salmonella* serovars (Singh et al., 2014), *E. coli* O157:H7 (Tang et al., 2014), and *Bacillus* spp. (Singh et al., submitted). However, these studies did not investigate if mathematical modeling could be used to explain resulting scatter patterns of colonies. Here we focused on developing and comparing the computational model for *B. subtitis* ATCC 6633 and *B. polymyxa* B719W for speckle formation and its resultant effect on the experimental scatter pattern. Such *in silco* study would be of great significance for extrapolating the colonial properties of highly virulent *Bacillus* species such as *Bacillus anthracis*, a causative agent of anthrax that requires a high containment facility for handling.

Rather than utilizing specific labeling reagents, proposed method adopted the combination of a laser light and a 2D CMOS sensor to transform the both 3-D macroscopic and microscopic structures (morphology) and material characteristics (refractive indices) effect into a single 2-D scatter image which can be further analyzed by chemometric methods. The proposed method does not disrupt the colony structure while capturing the scatter pattern, thus can be further used in biochemical, molecular, immunological and mass-spectroscopic methods for confirmation of the cultures.

As the modeling of forward scattering pattern for the bacteria colony shows, the morphology and optical characteristics play major roles into formation of diffraction patterns. Previous studies have shown that availability of nutrients and agar concentration (Bae et al., 2011), and storage condition of the agar plate (Mialon et al., 2012) possibly affect the distribution/propagation of cells within a colony and the resulting diffraction pattern. Furthermore, previous studies (Bae et al., 2007, 2010; Kim et al., 2013) indicate that the forward scattering patterns produced by BARDOT are circular symmetric or concentric circle with some radial spokes for most of the microorganisms (*E. coli*, *Listeria*, *Salmonella*, and *Staphylococcus*) studied.

The outcome of this study would be helpful in analyzing the growth characteristic and understanding of the colonial behavior on solid agar surface using visual, physical, mathematical and empirical models. The results will also help in development of better scatter signatures based classification algorithm for microbial detection, especially the pathogens and spoilage microbes relevant to food safety, food quality and food security.

## Conclusion

Here we report a theoretical modeling and experimental verification of the swarming growth characteristics of *Bacillus* species using optical scattering technology. To quantify the growth characteristics, PCM and ICMA along with BARDOT were utilized. Scalar diffraction theory coupled with new random phase component provided good agreement with experimental speckle pattern formation. The results indicate that as bacteria grow, their average speckle size decrease and the number of smaller speckle increases due to the swarming growth characteristics of bacteria within the colony.

### Conflict of interest statement

Euiwon Bae and Arun K. Bhunia are inventors of “System and method for rapid detection and characterization of bacterial colonies using forward light scattering” (US Patent No. 7465560), designated BARDOT, described in this review. The authors declare that the research was conducted in the absence of any commercial or financial relationships that could be construed as a potential conflict of interest.

## References

[B1] Bae E. Aroonnual A. Bhunia A. K. Hirleman E. D. (2011). On the sensitivity of forward scattering patterns from bacterial colonies to media composition. J. Biophotonics 4, 236–243 10.1002/jbio.201000051 20549773

[B2] Bae E. Bai N. Aroonnual A. Robinson J. P. Bhunia A. K. Hirleman E. D. (2010). Modeling light propagation through bacterial colonies and its correlation with forward scattering patterns. J. Biomed. Opt. 15:045001 10.1117/1.3463003 20799796

[B3] Bae E. Banada P. P. Huff K. Bhunia A. K. Robinson J. P. Hirleman E. D. (2007). Biophysical modeling of forward scattering from bacterial colonies using scalar diffraction theory. Appl. Opt. 46, 3639–3648 10.1364/AO.46.003639 17514326

[B4] Banada P. P. Guo S. L. Bayraktar B. Bae E. Rajwa B. Robinson J. P. (2007). Optical forward-scattering for detection of *Listeria monocytogenes* and other *Listeria* species. Biosens. Bioelectron. 22, 1664–1671 10.1016/j.bios.2006.07.028 16949268

[B5] Banada P. P. Huff K. Bae E. Rajwa B. Aroonnual A. Bayraktar B. (2009). Label-free detection of multiple bacterial pathogens using light-scattering sensor. Biosens. Bioelectron. 24, 1685–1692 10.1016/j.bios.2008.08.053 18945607

[B6] Bees M. A. Andresen P. Mosekilde E. Givskov M. (2000). The interaction of thin-film flow, bacterial swarming and cell differentiation in colonies of Serratia liquefaciens. J. Math. Biol. 40, 27–63 10.1007/s002850050004 10663662

[B7] Ben-Jacob E. Cohen I. Gutnick D. L. (1998). Cooperative organization of bacterial colonies: from genotype to morphotype. Annu. Rev. Microbiol. 52, 779–806 10.1146/annurev.micro.52.1.779 9891813

[B8] Cohen I. Golding I. Kozlovsky Y. Ben-Jacob E. Ron I. G. (1999). Continuous and discrete models of cooperation in complex bacterial colonies. Fractals 7, 235–247 10.1142/S0218348X99000244

[B9] Granek J. A. Magwene P. M. (2010). Environmental and Genetic determinants of colony morphology in *Yeast*. PLoS Genet. 6:e1000823 10.1371/journal.pgen.1000823 20107600 PMC2809765

[B10] Harshey R. M. (2003). Bacterial motility on a surface: many ways to a common goal. Annu. Rev. Microbiol. 57, 249–273 10.1146/annurev.micro.57.030502.091014 14527279

[B11] Henegariu O. Heerema N. A. Dlouhy S. R. Vance G. H. Vogt P. H. (1997). Multiplex PCR: critical parameters and step-by-step protocol. Biotechniques 23, 504–511 9298224 10.2144/97233rr01

[B12] Huff K. Aroonnual A. Littlejohn A. E. F. Rajwa B. Bae E. Banada P. P. (2012). Light-scattering sensor for real-time identification of *Vibrio parahaemolyticus*, *Vibrio vulnificus* and *Vibrio cholerae* colonies on solid agar plate. Microb. Biotechnol. 5, 607–620 10.1111/j.1751-7915.2012.00349.x 22613192 PMC3815873

[B13] Kaito C. Sekimizu K. (2007). Colony spreading in *Staphylococcus aureus*. J. Bacteriol. 189, 2553–2557 10.1128/JB.01635-06 17194792 PMC1899384

[B14] Kawasaki K. Mochizuki A. Matsushita M. Umeda T. Shigesada N. (1997). Modeling spatio-temporal patterns generated by *Bacillus subtilis*. J. Theor. Biol. 188, 177–185 10.1006/jtbi.1997.0462 9379672

[B15] Keyence-Corporation. (2006). Surface Scanning Laser Confocal Displacement Meter LT-9001 Series. Osaka: Keyence Corporation

[B16] Kim H. Bai N. Bhunia A. K. King G. B. Hirleman E. D. Bae E. (2013). Development of an integrated optical analyzer for characterization of growth dynamics of bacterial colonies. J. Biophotonics 6, 929–937 10.1002/jbio.201200224 23606315

[B17] Kozlovsky Y. Cohen I. Golding I. Ben-Jacob E. (1999). Lubricating bacteria model for branching growth of bacterial colonies. Phys. Rev. E 59, 7025–7035 10.1103/PhysRevE.59.7025 11969691

[B18] Kuo W. S. Chak K. F. (1996). Identification of novel cry-type genes from *Bacillus thuringiensis* strains on the basis of restriction fragment length polymorphism of the PCR-amplified DNA. Appl. Environ. Microbiol. 62, 1369–1377 8919799 10.1128/aem.62.4.1369-1377.1996 PMC167904

[B19] Lega J. Passot T. (2003). Hydrodynamics of bacterial colonies: a model. Phys. Rev. E 67, (3 Pt 1):031906 10.1103/PhysRevE.67.031906 12689100

[B20] Lorenz T. C. (2012). Polymerase chain reaction: basic protocol plus troubleshooting and optimization strategies. J. Vis. Exp. e3998 10.3791/3998 22664923 PMC4846334

[B21] Manzano M. Giusto C. Iacumin L. Cantoni C. Comi G. (2003). A molecular method to detect *Bacillus cereus* from a coffee concentrate sample used in industrial preparations. J. Appl. Microbiol. 95, 1361–1366 10.1046/j.1365-2672.2003.02120.x 14633011

[B22] Marcoux P. Dupoy M. Cuer A. Kodja J.-L. Lefebvre A. Licari F. (2014). Optical forward-scattering for identification of bacteria within microcolonies. Appl. Microbiol. Biotechnol. 98, 2243–2254 10.1007/s00253-013-5495-4 24413976

[B23] Merkus H. G. (2009). Particle Size Measurements: Fundamentals, Practice, Quality. Osaka: Springer Science & Business Media ISBN: 9781402090165. 10.1007/978-1-4020-9016-5

[B24] Mialon M. Tang Y. Singh A. K. Bae E. Bhunia A. K. (2012). Effects of preparation and storage of agar media on the sensitivity of bacterial forward scattering patterns. Open J. Appl. Biosens. 1, 26–35 10.4236/ojab.2012.13004

[B25] Nagai S. Nishizawa Y. Onodera M. Aiba S. (1971). Effect of dissolved oxygen on growth yield and aldolase activity in chemostat culture of *Azotobacter vinelandii*. J. Gen. Microbiol. 66, 197–203 10.1099/00221287-66-2-197 4937139

[B26] Ngamwongsatit P. Banada P. P. Panbangred W. Bhunia A. K. (2008). WST-1-based cell cytotoxicity assay as a substitute for MTT-based assay for rapid detection of toxigenic *Bacillus* species using CHO cell line. J. Microbiol. Methods 73, 211–215 10.1016/j.mimet.2008.03.002 18417231

[B27] Pipe L. Z. Grimson M. J. (2008). Spatial-temporal modelling of bacterial colony growth on solid media. Mol. Biosyst. 4, 192–198 10.1039/b708241j 18437261

[B27a] Proctor R. A. von Eiff C. Kahl B. C. Becker K. McNamara P. Herrmann M. (2006). Small colony variants: a pathogenic form of bacteria that facilitates persistent and recurrent infections. Nat. Rev. Microbiol. 4, 295–305 10.1038/nrmicro1384 16541137

[B28] Shapiro J. A. (1992). Pattern and control in bacterial colony development. Sci. Prog. 76, 399–424 1364579

[B29] Shimada Y. Nakahara A. Matsushita M. Matsuyama T. (1995). Spatiotemporal patterns produced by bacteria. J. Phys. Soc. Jpn. 64, 1896–1899 10.1143/JPSJ.64.1896

[B30] Singh A. K. Bettasso A. M. Bae E. Rajwa B. Dundar M. M. Forster M. D. (2014). Laser optical sensor, a label-free on-plate *Salmonella enterica* colony detection tool. mBio 5:e01019-13 10.1128/mBio.01019-13 24496794 PMC3950520

[B32] Stecchini M. L. Del Torre M. Donda S. Maltini E. Pacor S. (2001). Influence of agar content on the growth parameters of *Bacillus cereus*. Int. J. Food Microbiol. 64, 81–88 10.1016/S0168-1605(00)00436-0 11252514

[B33] Suchwalko A. Buzalewicz I. Wieliczko A. Podbielska H. (2013). Bacteria species identification by the statistical analysis of bacterial colonies Fresnel patterns. Opt. Express 21, 11322–11337 10.1364/OE.21.011322 23669989

[B34] Tang Y. Kim H. Singh A. K. Aronnual A. Bae E. Rajwa R. (2014). Light scattering sensor for direct identification of colonies of Escherichia coli serogroups O26, O45, O103, O111, O121, O145 and O157. PLoS ONE 9:e105272 10.1371/journal.pone.0105272 25136836 PMC4138183

[B35] Vliet L. J. V. (2014). DIPimage and DIPlib Ver. 2.6. [Online]. Available online at: http://www.diplib.org/main

[B36] Wimpenny J. W. T. (1992). Microbial systems—patterns in time and space. Adv. Microb. Ecol. 12, 469–522 10.1007/978-1-4684-7609-5_10

